# Supplementary figures and images for: Tumor Intrinsic Immunogenicity Suppressor SETDB1 Worsens the Prognosis of Patients with Hepatocellular Carcinoma
Source: Cells. 2024 Dec 19;13(24):2102. doi: 10.3390/cells13242102 (PMC11675013; doi:10.3390/cells13242102)

## Supplementary

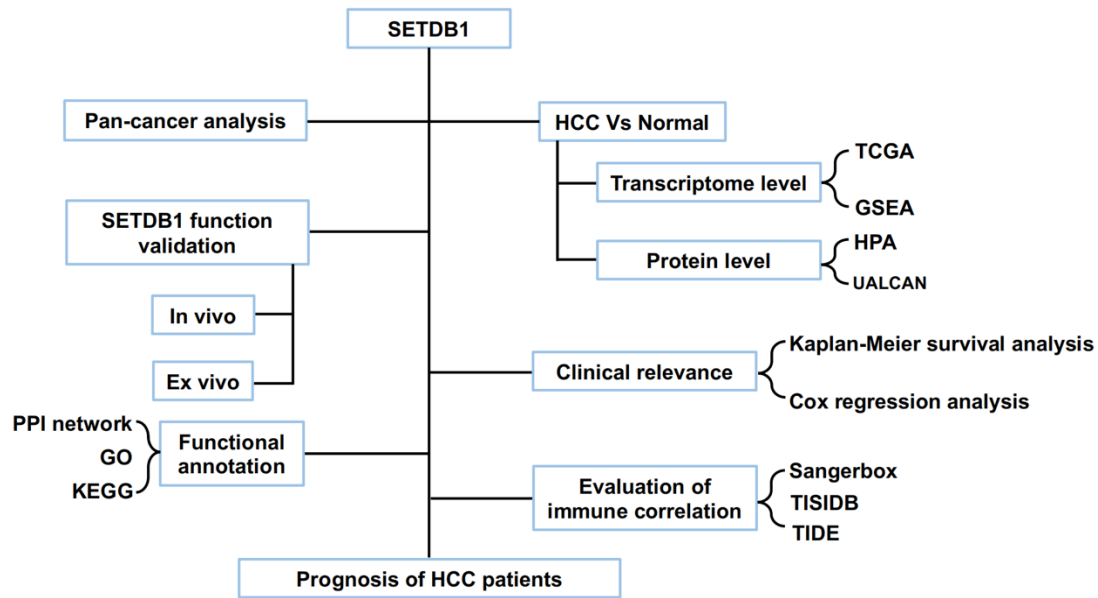

**Figure S1.** The flowchart. This figure illustrates the workflow of our study.

Supplement: Supplementary file 1 [file cells-13-02102-s001.zip › cells-3292618-Figure S1.pdf]
